# Present and future potential of plant-derived products to control arthropods of veterinary and medical significance

**DOI:** 10.1186/1756-3305-7-28

**Published:** 2014-01-15

**Authors:** David R George, Robert D Finn, Kirsty M Graham, Olivier AE Sparagano

**Affiliations:** 1 Department of Applied Sciences, Faculty of Health and Life Sciences, Northumbria University, Newcastle upon Tyne NE1 8ST, UK

**Keywords:** Botanical, Phytochemical, Plant-derived product, Insecticide, Acaricide, Pesticide, Repellent

## Abstract

The use of synthetic pesticides and repellents to target pests of veterinary and medical significance is becoming increasingly problematic. One alternative approach employs the bioactive attributes of plant-derived products (PDPs). These are particularly attractive on the grounds of low mammalian toxicity, short environmental persistence and complex chemistries that should limit development of pest resistance against them.

Several pesticides and repellents based on PDPs are already available, and in some cases widely utilised, in modern pest management. Many more have a long history of traditional use in poorer areas of the globe where access to synthetic pesticides is often limited. Preliminary studies support that PDPs could be more widely used to target numerous medical and veterinary pests, with modes of action often specific to invertebrates.

Though their current and future potential appears significant, development and deployment of PDPs to target veterinary and medical pests is not without issue. Variable efficacy is widely recognised as a restraint to PDPs for pest control. Identifying and developing natural bioactive PDP components in place of chemically less-stable raw or 'whole’ products seems to be the most popular solution to this problem. A limited residual activity, often due to photosensitivity or high volatility, is a further drawback in some cases (though potentially advantageous in others). Nevertheless, encapsulation technologies and other slow-release mechanisms offer strong potential to improve residual activity where needed.

The current review provides a summary of existing use and future potential of PDPs against ectoparasites of veterinary and medical significance. Four main types of PDP are considered (pyrethrum, neem, essential oils and plant extracts) for their pesticidal, growth regulating and repellent or deterrent properties. An overview of existing use and research for each is provided, with direction to more extensive reviews given in many sections. Sections to highlight potential issues, modes of action and emerging and future potential are also included.

## Introduction

The use of synthetic products to treat pests of veterinary and medical significance is becoming increasingly problematic. Issues including pest resistance, product residues, withdrawal of active ingredients, undesirable environmental persistence and unacceptable risks to non-target organisms are among those driving research to identify alternative approaches. One approach employs the repellent, toxic or otherwise bioactive effects of plant-derived products (PDPs). These products are attractive as pesticide candidates on the grounds of generally low mammalian toxicity, short environmental persistence and, for many, complex chemistries that should limit development of pest resistance against them [1].

Several types of PDP can be distinguished from the literature. In his 2006 review on the use of botanicals in agriculture Murray Isman identified four major types of PDP used for insect control: pyrethrum, rotenone, neem, and essential oils [2], though rotenone has since been widely withdrawn from use as a result of stricter legislation on pesticide actives being enforced in many countries. Pyrethrum, neem and essential oils, however, are all in widespread use – commercially or in empirical studies – to target pests of veterinary and/or medical significance. In some sectors the use of plant extracts is also popular (see Pesticidal potential).

The remainder of this review focuses on the pesticidal, developmental and repellent potential of PDPs, potential issues with their use, their modes of action, and their emerging and future potential in treatment and prevention of veterinary and medical ectoparasitoses. With the bank of literature on PDP potential against veterinary and medical ectoparasites increasing considerably in recent years we do not aim to exhaustively review the subject area, instead directing the reader to more extensive reviews where appropriate (Table 1). Rather we present an overview of work in this burgeoning field, with reference to major PDP types and pest groups, to allow general but informed conclusions to be made on the realised and future potential of PDPs against veterinary and medical pests. Consideration of the literature on endoparasites and PDPs is beyond the scope of this work, though reviews covering this subject in full or in part can be accessed elsewhere for both PDPs in general and specific product types [3,4]. To provide continuity and demonstrate the broad potential of PDPs against any given pest, *Dermanyssus gallinae* (the poultry red mite) is regularly referred to throughout as an exemplar species, with most sections also featuring work with ticks and mosquitoes.

**Table 1:** Selected reviews covering the use of PDPs against pests of veterinary, medical and agricultural significance

**Author/s**	**Year**	**Focal PDP/s**	**Focal mode/s of action**	**Focal target/s**	**Focal host/s group/s**
Casida	1980	Pyrethrum	Toxicant; Repellent; Knockdown	Insects	Veterinary; Medical
Schmutterer	1990	Neem	Numerous	Numerous	Medical; Agricultural*
Sukumar *et al*.	1991	Numerous	Numerous	Mosquitoes	Medical
Mulla and Su	1999	Neem	Numerous	Ectoparasites	Veterinary; Medical
Brahmachari	2004	Neem	Numerous	Numerous, including pathogens* and endoparasites*	Medical; Agricultural*
Shaalan *et al*.	2005	Essential oils	Larvicide; Insect Growth Regulator	Mosquitoes	Medical
Isman	2006	Numerous	Antifeedant; Repellent; Toxicant	Insects	Medical; Agricultural*
Bakkali *et al*.	2008	Essential oils	Cytotoxicity including cell membrane damage	Numerous, including pathogens*	Medical; Agricultural*
George *et al*.	2008	Multiple	Toxicant; Repellent	Ectoparasites	Veterinary
Koul *et al*.	2008	Essential oils	Numerous	Numerous	Medical; Agricultural*
Russo *et al*.	2009	Numerous	Toxicant; Repellent	Numerous, including endoparasites*	Veterinary
Bissinger and Roe	2010	Focus on Terpenoids	Repellent	Ticks	Medical; Veterinary
Nerio *et al*.	2010	Essential oils	Repellent	Mosquitoes	Medical
Schmahl *et al*.	2010	Essential oils, Neem	Toxicant; Repellent	Ectoparasites	Veterinary; Medical; Agricultural*
Maia and Moore	2011	Numerous	Repellent	Mosquitoes	Medical
Ogbuewu *et al*.	2011	Neem	Numerous	Numerous	Veterinary; Medical; Agricultural*
Regnault-Roger	2011	Essential oils	Toxicant	Insects	Agricultural; Medical
Saha *et al*.	2011	Neem	Repellent	Insects	Agricultural*
Ghosh *et al*.	2012	Extracts	Numerous	Mosquito (larvae)	Medical
Kiss *et al*.	2012	Multiple	Toxicant; repellent	Ticks	Veterinary

*Not covered by the current review.

## Review

### Pesticidal potential

Several pesticides based on PDPs are already available, and in some cases widely utilised, in modern pest management [2,5-7]. Numerous proof of concept studies support that additional PDPs could be of further use, including targeting numerous veterinary and medical pests. Many more have a long and continuing history of use in poorer areas of the globe where access to synthetic pesticides is often limited. However, though such use may greatly assist researchers in identifying novel PDPs for further testing [8,9], we do not consider traditional use to necessarily confirm efficacy. Hence, examples that only report on such use without further study have not been included.

#### Pyrethrum

Technical grade pyrethrum is extracted from dried and ground flowers of the daisy *Tanacetum cinerariaefolium* and typically contains 20-25% pyrethrins (I and II) as its main pesticidal components [2]. Use of pyrethrum to treat pests of veterinary and medical significance greatly pre-dates the advent of synthetic 'second generation’ pesticides [10], to which pyrethroids (synthetic modifications of pyrethrins that remain in widespread use to the present day) belong. Pyrethrum remains in widespread use to the present day, with its current contribution to veterinary and medical pest management being primarily in the treatment of premise pests, such as cockroaches and flies, which could serve as disease vectors.

The pesticidal potential of pyrethrum was apparently recognised in the 17th Century, though verification is reported to have occurred later in 1840 [11]. Following verification, pyrethrum was rapidly adopted as a household insecticide, also being incorporated into mosquito control as the primary active component of sticks/coils. Issues regarding photostability limit the use of pyrethrum outdoors (see Potential issues) and this drove the development of synthetic pyrethroids in the mid-1900s. Though the advent of pyrethroids led to dramatic declines in pyrethrum use, the latter remains popular where product safety is paramount (e.g. organic production, residential use and use in food handling premises) [11].

It has been reported that resistance is less likely to be developed to the natural *vs.* synthetic product [12], with resistance to pyrethroids now widely reported in numerous pest groups [11], (it should be noted, however, that this does not mean that resistance to natural compounds cannot develop; see Emerging and future potential). In work with *D. gallinae*, for example, permethrin tested at 100% only led to complete *in vitro* mortality of mites collected from 1 in 6 farms, with mortality as low as 30% for two of the farms tested [13]. Such resistance does not appear to effect pyrethrum toxicity, however, with work in mosquitoes supporting that pyrethrum may be effectively used to target pyrethroid-resistant *Anopheles gambiae*[14]. This latter finding, and the range of synthetic pyrethroids that have been developed/used in the past century, raises the possibility that the lack of resistance seen to the natural product may be as much a consequence of its low level of use in comparison to pyrethroids as its natural status [12]. This limited use may be attributable to the low photostability of pyrethrum, despite the fact that knock-down with pyrethrum is generally rapid [2].

#### Neem

The neem tree (*Azadirachta indica*) has a long history of traditional use in its country of origin (India). Subsequent research has revealed a vast and diverse chemistry to this species, including within this the triterpene azadirachtin to which much of the pesticidal activity of neem is attributed [15]. Most neem-based products are formed from seed extracts, where azadirachtin naturally occurring at relatively low levels (<1%) is concentrated to 10-50% to form technical grade material [2].

Neem seed extract is known to display activity to a vast range of pest invertebrates [16], including against a multitude of pests of veterinary and medical significance [17,18]. Consequently, it is of no surprise that many neem-based products are commercially available for use in these sectors. Tre-san®, MiteStop®, Wash Away Louse® and Picksan LouseStop®, all based on neem seed extracts, are typical examples resulting from the work of one research group alone [18]. These products have been developed for use against a range of veterinary and medical pests including dust mites, ticks, ectoparasitic mites (including *D. gallinae*), scabies mites (*Sarcoptes scabiei*) and head lice (*Pediculus humanus capitis*). Such products may be highly efficacious, with MiteStop® reported to be more effective against *D. gallinae* than the synthetic organophosphate phoxim [19]. This same group has recently shown that a similar product (Licener®) can be highly efficacious against the human head louse [20], as are comparable products based on work elsewhere [21]. NeemAzal® is another example of a commercial neem-based product available in several countries, in this case to target ticks [22].

It deserves note that the above studies were performed under laboratory conditions and the effects observed were dependant on exposure methodologies. In the laboratory, for example, a 1:40 dilution of MiteStop® killed *Ixodes* ticks irrespective of the mode of contact, but only direct spraying on the backs of *Rhipicephalus* ticks had a significant effect [18]. Though such laboratory data do not necessarily translate to field effectiveness, studies in livestock have now confirmed *in vivo* tick potential [22], with topical application proving successful in cattle [23,24] and oral administration in lambs suggesting future potential for administration in feed [25]. In the studies performed in cattle, it was confirmed that the seed extract had the highest acaricidal effect, where in addition a significant reduction in egg laying potential was observed in ticks that survived treatment. In comparison to the synthetic pyrethroid, cypremethrin, neem seed extract was found to be comparable in terms of reducing oviposition, despite having a longer period to knockdown (5 days as opposed to 3) [24]. Oral administration to lambs was found not to result in lethality to *Dermacentor vairiabilis* ticks, though based on a significant reduction in tick weight the authors concluded that the presence of azadirachtin in the host’s blood interfered with tick feeding. The authors speculated that oral administration may have a greater effect on one-host tick species, though this has yet to be tested [25].

Bioactivity of neem-based products is not limited to seed extracts and other preparations may also be of use. In work with ixodid ticks, for example, 70% mortality was achieved following exposure to a methanolic bark extract [26]. Cold presses from neem seeds (syn: neem oils) are also reported to be effective against mites and soft-bodied insects [2], with use of cardboard traps impregnated with 20% neem oil reducing *D. gallinae* populations *in vivo* by more than 90% [27]. Various formulations based on neem, such as those in water and methyl-tert-butylether/water, may also be of use in mosquito control, particularly as larvicides or growth regulators/inhibitors [17,28].

Neem has been reported to retain its pesticidal efficacy relatively well, even against pests renowned for development of resistance (including to other biological insecticides such as *Bt* and spinosad). Laboratory study has shown, for example, that even after 42 generations, diamondback moth (*Plutella xylostella*) larvae were still susceptible to neem oil [29]. Neem also has a history of safe use as an insecticide. Though negative impacts on mammals may result from sub-acute or chronic exposure to neem, a review of these balanced against likely ingestion of neem residues concluded that its use as a pesticide “should not be discouraged” [30].

#### Essential oils and other plant extracts

Essential oils are naturally produced by plants as secondary compounds, being obtained for commercial use by various forms of distillation. They are notably diverse in terms of chemistry, containing up to 60 separate components (per oil) primarily composed of terpenes, terpenoids and other aromatic compounds [31]. Plant extracts on the other hand are obtained through various forms of solvent extraction, as for many neem formulations and pyrethrum above, with or without subsequent purification and/or separation/concentration/drying as required. Distillation is based on separation via differences in volatility and requires heating the liquid to the boiling point which is specific for each substance. Distillation methods can include: water or steam distillation; rectification; cohobation; fractional distillation and percolation. Extraction methodologies, typically used for botanical material that has a low yield of essential oils, include: maceration; enfleurage; supercritical CO^2^; expression (cold press extraction) and solvent free microwave extraction.

Work tends to either focus on commercially available products from the flavour/perfume industry, for example against *D. gallinae*[32], or lab-prepared products often sourced from novel wild-growing plant material, for example against mosquitoes [8,9,33-36] or head lice [37,38]. Though the literature on the subject has been dominated with proof of concept 'screening’ studies, as the potential of oils of extracts has been recognised more work has been devoted to investigating product chemistries and active components (see Potential issues), as well as disentangling modes of action at both the mechanistic [39,40] and bio-molecular (see Modes of action) level.

Due to their high chemical diversity and consequent potential for bioactivity, essential oils have received significant interest from scientists seeking novel and natural alternatives to synthetic pesticides in all sectors, including agriculture, veterinary/medical medicine and apiculture [2,5,7]. An extensive account of research into essential oils for pest control *per se* is given by Bakkali *et al*. [31], where the potential of these products to target viruses, fungi, bacteria and protozoa is also covered in depth. In addition to general works on essential oil pesticidal potential, authors have also presented reviews focusing on key target groups, particularly mosquitoes [28,41,42].

George *et al.*[5] reviewed the potential of both essential oils and extracts to a range of ectoparasites of veterinary significance, citing examples of their use against pests of livestock, poultry and domestic animals. Amongst the examples given, 100% mortality was achieved for *Psoroptes cuniculi* with a floral extract of chamomile [43], and for *D. gallinae* with numerous essential oils [44], as later supported in the authors’ own work [32]. In a study employing extracts from indigenous Indian plants, Zahir *et al.*[9] similarly showed that products could be of use in targeting multiple ectoparasites of veterinary and medical significance, in this case *Rhipicephalus (Boophilus) microplus* ticks and the larvae of *Anopheles subpictus* and *Culex tritaeniorhynchus*. Examples of the successful use of several commercial PDP-based products to target fleas, ticks and mange mites in cats and dogs were also cited, as were cases involving ectoparasitic mites infesting honeybees [5]. More recently, Kiss *et al*. [22] devoted multiple sections of their review on tick prevention to botanicals, noting research undertaken with both essential oils and extracts targeting larvae and adults alike. Importantly, the authors highlighted the potential influence of factors such as solvent selection and the life-stage of the target organism on efficacy. Younger stages were typically more susceptible than adults [22], as has also been reported for *D. gallinae* where higher toxicity of essential oils to juvenile (*vs.* adult) mites was recorded for cade, clove bud and garlic [45]. To highlight the importance of solvent selection, the authors cited work in which ethyl acetate root extracts of *Senna italica* were effective against *Hyalomma marginatum rufipes*, whilst extracts derived from numerous other solvents were not [46].

Essential oils and extracts have also been investigated to target pests of medical significance. Studies with mosquitoes and these products are particularly abundant and weighted towards development of repellents (see Repellent potential) and larvicides; potentially for combined use in an IMM (Integrated Mosquito Management) approach. Pitasawat *et al*. [47], for example, tested five essential oils for larvicidal activity against *Anopheles dirus* and *Aedes aegypti*. All products killed larvae in 24 hr toxicity tests, with the authors citing separate studies in which basil, cinnamon, citronella and thymus essential oils showed similar promise [47]. Ghosh *et al*. [42] produced a recent review of plant extracts as mosquito larvicides, presenting an extensive list of products, including octacosane, germacrene D and azadirachtin, shown to be toxic to multiple species. In all the authors tabulated 186 examples of PDP toxicity to mosquitoes (extract type x plant species x mosquito species), adding to previous reviews by Shaalan *et al*. [28] and Sukumar *et al*. [41] covering the same subject. These earlier reviews also suggest that many extracts can play a role in IMM as growth/reproduction inhibitors or oviposition deterrents [28,41].

Various lice and scabies mites have also received interest as targets for essential oils and extracts, this being at least partially encouraged by consumer health concerns regarding topical synthetics and the resulting search for alternatives. In work by Oladimeji *et al*. [48], the authors tested the essential oil of *Lippia multiflora* against body lice, head lice and scabies mites, with knock-down and overall efficacy exceeding that of the standard synthetic treatments tested. Several products based on oils and/or extracts are already commercially available, especially for head lice. Scientific scrutiny of such products often suggests that 'field’ efficacy may be questionable [49,50], with similar conclusions drawn for commercial repellents aimed at the same pest (see Repellent potential). Nevertheless, some products do appear effective, with Tea Tree Gel® outperforming 1% permethrin in an Australian study [50]. Commercial extracts have also found a place in poultry pest management with approval of Breck-a-Sol®, a garlic-based acaricide, for use against *D. gallinae*[5].

### Repellent potential

Studies considering PDPs as repellents tend to focus upon essential oils and their bioactive constituents, for the probable reason that high volatility of these products lends itself well to such work. To date, research into the repellent potential of PDPs has been dominated by work targeting biting flies of medical significance, particularly mosquitoes, with the bulk of research and development in this field concentrated on topical repellents [51,52]. The over-riding aim of this work is to generate alternatives to DEET, which though effective carries a health risk, particularly for infants [2]. In many cases PDPs have been found to compare well to synthetic repellents in terms of short-term repellence, though effects tend to be short-lived and comparable longer-term repellence is harder to achieve. 0.1 ml undiluted clove oil per 30 cm^2^ skin has, for example, been found to provide excellent repellence to *Aedes aegypti*, *Culex quinquefasciatus* and *Anopheles dirus*, being 100% effective if only for 2–4 hr [53]. Neem-based preparations have been found to offer protection from mosquito species for a similar period, but with reduced efficacy at a rate of 2 g/person [15] and variability between studies [51]. In contrast, 100% DEET may provide reliable protection for 10 hrs or more, depending upon the formulation used and environmental variables experienced post-application. DEET may not be readily accessible to inhabitants of poorer communities, however, and in these instances identifying locally available plants with repellent properties against a range of mosquito species has obvious advantages [54].

Several PDPs are already widely used as insect repellents, despite their current requirement for relatively frequent re-application. Indeed, the US Environmental Protection Agency has registered citronella, lemon and eucalyptus essential oils as insect repellent ingredients for topical use [52]. Many more examples of the use of essential oils and their chemical constituents as repellents against mosquitoes are provided in recent reviews by Maia and Moore [51] and Nerio *et al*. [52]. In the latter work examples are also provided for numerous other pest Orders of veterinary, medical and agricultural interest, particularly beetles.

After mosquitoes it seems that most work on PDPs as repellents for pests of veterinary and medical significance has centred on ticks, often with encouraging results [22,55]. The livestock brown ear tick, *Rhipicephalus appendiculatus*, for example, was repelled just as effectively by *Gynandropsis gyandra* essential oil at high dose as by DEET when tested using the tick climbing assay [56]. Essential oils of citronella, clove and lily of the valley were similarly effective in comparison to DEET when tested against *Ixodes ricinus*[57], as was diluted rhododendron oil when tested against the same species *in vitro* and *in vivo* (via a blanket-dragging method) [58].

The repellent properties of PDPs to other pest groups have also been investigated, albeit not as extensively. George *et al*. [59], for example, tested the repellence of seven essential oils to *D. gallinae*. All showed at least short-term repellence and that of thyme lasted for the entire 13 day study duration [59]. Birkett *et al*. [60] tested the repellence of catmint essential oil and its main iridoid compounds to *D. gallinae* as well as ticks (*R. appendiculatus*) and multiple mosquito species. The results demonstrated general repellence to all groups, though the authors reported variable results across mosquito species and improved activity of whole essential oils over isolated or simple mixes of their compounds. Finally, work with permethrin-resistant head lice has shown certain essential oils from Argentinian plants, and their components, to be effective repellents [37,38]. The most effective essential oils included those extracted from *Myrcianthes cisplatensis* and *Mentha pulegium*[37] and *Cinnamomum porphyrium* and *Aloysia citriodora* (Type 2) [38], with the most effective components within these oils being 1,8-cineole, anisole and benzyl alcohol. While these are encouraging and promising findings, in work that compared commercially-available PDP-based repellents to DEET for *in vitro* repellence to head lice, neither were found to be effective [61].

### Potential issues

Development and deployment of PDPs to target medical and veterinary pests is not without issue, where variable efficacy is perhaps the most significant restraint. Variations in product chemistries, caused by any combination of a number of post- and pre-harvest biotic and abiotic factors, may result in dramatically altered bioactivity of the 'end product’ to target pests [2,22,28,42]. Chemical variation has, for example, been reported between essential oils from different varieties [62,63], parts [63,64] and geographic locales [65,66] of a single plant species, with seasonality [67,68], method of oil extraction [69], year of harvest [70] and storage conditions [70] also influencing essential oil chemistry. In work with *D. gallinae* variable efficacy of essential oils has been reported within a single study [39], with comparison of results from this work to independent research [44], suggesting similar variability between studies concerning the toxicity of pennyroyal to this pest. However, analysis of essential oils suggests that some plants appear less likely to vary their chemical profiles in response to environmental changes than others, even within a single species, as illustrated in work carried out on *Thymus pulegioides*[71]. Careful strain selection may thus, at least partially, overcome this issue, even for highly chemically diverse products like essential oils.

Identification of individual bioactive PDP components for use in place of chemically less-stable 'raw’ or 'whole’ products may also be a solution to variable efficacy [72]. Neem, for example, is manufactured based on azadirachtin content providing some stability in technical grade products. Terpenes such as thymol (found in high concentrations in thyme essential oil amongst others) may offer a similar solution for essential oils. Thymol has shown significant potential against multiple ectoparasitic mites of veterinary significance (along with other terpenes); with several thymol-based commercial formulations widely used in apiculture to target bee mites [5]. Eugenol has shown similar promise against scabies mites, providing comparable toxicity to benzyl benzoate and killing mites within 1 hour under laboratory conditions [73]. Terpenes have also been considered for their repellent potential. Both terpineol and 1,8-cineole (3% in olive oil), for example, offered complete protection from *Culex pipiens molestus*[74]. Nevertheless, some studies report improved toxicity [1] or repellence [60] of whole essential oils *vs*. simple mixtures of their component chemicals, supporting use of the former for optimal efficacy. Diverse blends of individual components to effectively create artificial 'essential oils’ may offer a best-of-both-worlds solution, potentially allowing products to be tailored to pests. Whether or not such a product could still be considered 'natural’ may, however, be questionable. A recent study has also illustrated the importance of other components used in product formulation, with a simple change in the type of alcohol used (isopropanol for ethanol) having a significant effect on the repellency of geraniol based products to *Amblyomma americanum* in both laboratory and field-based tests [75], just as solvent selection may effect PDP toxicity (see Essential oils and other plant extracts). Stability of essential oils post-processing whilst in storage may also present an issue, though one beyond the scope of the current review and recently considered elsewhere [76].

Short residual activities are a further drawback for many PDPs. Pyrethrum and neem, for example, are both relatively highly UV-degradable [2]. The high volatility of essential oils has a similar effect on their residual activity as both pesticides and repellents. George *et al*. [77], for example, demonstrated mortality in *D. gallinae* of up to 90% after exposure to arenas treated with lavender essential oil minutes before adding mites. Where mites were not added to arenas for 24 hrs post-treatment, however, mortality fell to nearer 10%. The residual activity of terpenes is often similarly limited. In the aforementioned work by Traboulsi *et al*. [74], for example, terpenes only offered protection from mosquito bites for a maximum of 2 hrs, comparing poorly with synthetics as a result (see Repellent potential). Nevertheless, slow-release technologies are being increasingly developed to prolong the bioactivity of PDPs, suggesting that limited residual efficacy should pose an ever-decreasing problem in the future. In their recent review of PDPs as mosquito repellents, Maia and Moore [51] cite examples of both encapsulation and microencapsulation as a means to prolong essential oil repellence, with the latter being used to increase efficacy of citronella-treated fabric for up to 30 days [78]. Saha *et al*. [79] have similarly reported on the potential benefits of microencapsulation, inclusion complexes, microemulsions and granular formulations to enhance efficacy and shelf life of neem-based products.

Where pesticides are concerned, in some instances short persistence times may even be considered advantageous [22]. As long as rapid knock-down can be achieved, which seems the case for at least some PDPs such as pyrethrum [2], essential oils [80] and terpenes [73], low residual activity should infer minimal impact on non-target organisms and the environment, also supporting optimally short product withdrawal periods where relevant. Rapid knock-down cannot be assumed universal for PDPs, however, with extracts (including neem) noted as being comparatively slow in exerting their full effect [22]. Prolonging the persistence times of PDPs may raise new issues regarding their environmental toxicity. Half-lives of most essential oil based products, for example, are currently <24 hrs on treated surfaces and in soil/water [81], allowing rapid breakdown of active ingredients and consequent minimal effects on non-target organisms, at least in comparison to longer lasting synthetics. PDPs may exert broad spectrum effects on groups such as insects, however, and prolonged persistence times may lead to increased exposure to non-target species, including beneficials (e.g. pollinators and pest natural enemies). Ensuring that developments in extending PDP persistence progress without compromising their generally favourable environmental profile is an important challenge for future work in this field.

Though generally considered safe for mammals, some PDPs have been shown to exert negative health and welfare effects in humans and other animals. As noted in Background, for example, the PDP rotenone is no-longer widely available as a pesticide, having been withdrawn from markets due to health and environmental concerns associated with its use. Multiple studies have, for example, linked rotenone to Parkinson’s Disease [82,83]. Even seemingly innocuous products, such as essential oils, may invoke negative responses at sufficient concentrations or in certain vertebrates. In work with laying hens, for example, birds were found to tolerate high exposure to thyme essential oil without incident, but became lethargic, depressed and unproductive when exposed to pennyroyal [84]. Indeed, certain botanicals that exert their effect on insect nervous systems (see Modes of action), may be relatively toxic to birds, fish, reptiles and amphibians [28]. It is also reported that commercial flea products containing essential oils may have negative effects on companion animals, with cats in particular being unable to metabolise these products due to an inability to glucoronidate [85]. In extreme cases death of companion animals has been recorded following exposure, though responses are typically less severe (e.g. agitation, tremors, lethargy) [85]. Further examples of deleterious effects of various PDPs in domestic animals are given by Russo *et al*. [3], where increased emphasis is given to orally administered products. Evidence such as this dispels the common misconception that all PDPs can be considered “safe” to vertebrates, though this may hold true in many cases [7], albeit with some 'purified’ products such as terpenes being more generally toxic than their parent material [86].

Despite their general non-toxicity to vertebrates, PDPs may exert broad-spectrum effects on invertebrates, including some non-target beneficial species. Reduced pupal emergence has been reported in predatory lacewings fed upon prey that had consumed neem oil [87], for example, with direct toxicity to *Macrolophus caliginosus* (a predatory mirid bug) also reported for neem formulations at lower than field rates [88]. Invertebrate selectivity is perhaps of greater concern when deploying PDPs over vast open areas in an agricultural setting, though should still be considered important in deployment against veterinary and medical pests, especially where release into the wider environment (e.g. mosquito repellents) or co-deployment with invertebrate-based biological control (e.g. for *D. gallinae* control) are factors. Fortuitously, research supports that specificity may be dependent upon the type of PDP and target pest under consideration, suggesting that some PDPs can display (at least relative) pest selectivity. Neem seed extract, for example, has been reported as generally safe for pollinators [89] and many pest natural enemies [90], despite being effective against 413 insect species *per se*[16]. Essential oils may also exert a stronger effect on some invertebrate groups than others [45], or on different members of the same pest group [42,91], suggesting similar potential for selectivity.

Other potential drawbacks of PDPs include sustainability of the botanical resource, regulatory approval and industry confidence in the product [2]. However, we consider these to be of secondary importance to reliability, residuality and non-target toxicity, where resolving these 'primary concerns’ must necessarily take priority to support regulatory approvals and end-user confidence in commercial products. This must additionally be achieved in a cost-effective manner if PDP-based products are to be more widely adopted commercially.

### Modes of action

For those PDPs with a long history of use, modes of action are relatively well established. Pyrethrum, for example, is known to act in much the same way as DDT; attacking sodium channels and serving as a neurotoxin. Knock-down is subsequently relatively fast for pyrethrum, particularly so in flying insects [2,10]. The effects exerted by neem are also well known and primarily attributed to feeding deterrence and disruption to growth, though oviposition deterrence, repellence, reduced fitness and sterility may also result where neem is used [4,79,92]. In terms of its mode of action, neem has been shown to target the cholinergic system in insects through inhibition of acetylcholinestrase (AChE), and is also reported to disrupt hormonal balance [93]. Work with insect cell lines has further shown that azadirachtin (the main chemical component of neem) may exert an anti-mitotic effect by disrupting tubulin polymerisation [94]. More recent work in mammalian cell lines has revealed that azadirachtin may also cause cell-cycle arrest via down-regulation of cyclin B and cyclin D1, but also an induction of pro-apoptotic signals; a mode of action that relates to its insecticidal properties [95].

In contrast, relatively less has been done to establish modes of action in more novel PDPs with pesticidal potential, despite the wealth of research on the potential of essential oils and extracts in pest control [85]. The modes of action for many of these PDPs have been described in a general manner with toxic, antifeedant, antioviposition and developmental effects recorded *per se*[86]. This may, at least in part, result from many PDPs being classed as 'minimum risk pesticides’ according to the United States EPA. Products meeting the criteria of 'minimum risk’ (i.e. being 'demonstrably safe’) do not require EPA registration, negating the need for extensive data on modes of action to be collated prior to their commercialisation, at least in the US marketplace (though stricter legislation regarding the use of the same PDPs exists elsewhere where such 'exemption lists’ are not implemented). Nevertheless, those studies that have been conducted on the mode of action of essential oils suggest that these products can attack pests in a variety of ways [93]. Not all essential oils and/or terpenes are effective, however, and subtle structural differences in products may have large effects on their toxicity. In work with eugenol and iosoeugenol, for example, toxicity to the aphid *Aphis craccavora* varied according to product [96], despite the only difference being an alteration to the position of the double bond in the C6–C3 system. The authors speculated that the position of the double bond could be important in tempering mitochondrial respiration (or some other target) efficiently [96].

For those products that are effective, recent reviews covering various veterinary, medical and agricultural pests report essential oils and their constituents to primarily act upon on GABA, tyramine and octopamine receptors/synapses, and the inhibition of AChE [7,93,97]. In work with mosquitoes, the essential oil of *Psoralea corylifolia* has been reported to display genotoxic effects, through either direct interaction with DNA or by generation of DNA-damaging reactive oxygen species [98]. Disruption to transient receptor potential channels may be a further mode of action for numerous terpenoids, where, depending upon the product, exposure may result in activation or inhibition [97]. Whereas some terpenes may have opposing effects on a single system, others may act on several systems. Thymol, for example, has been reported to both block octopamine receptors and act upon the GABA-gated chloride channel [93]. Whatever the specific mode of action, lipophilicity of essential oils and their constituents may play an important role in efficacy, optimising penetration of products into the arthropod body [99]. The volatile nature of essential oils and their constituents may also optimise pest exposure to product. In independent studies with *D. gallinae*, for example, numerous essential oils including thyme, coriander and clove bud have been shown to exert fumigant toxicity [39,44]. For a reclusive pest like *D. gallinae* the ability of a product to penetrate refugia in which mites spend the majority of their time could be highly advantageous [100]. It can be surmised that other pests with comparable life-history traits (e.g. bedbugs) may be similarly suited to treatment with highly fumigant PDPs. When considering modes of action it is pertinent to also consider potential detoxification pathways as it may not be the parent compound but a metabolite that is elucidating the toxicity.

### Emerging and future potential

A long list of potentially pesticidal PDPs exists and this list will continue to grow in the future. However, in order to harness these products to their full potential there are several avenues that have to be explored further, primarily concerning the modes of action of these products (or the active components they contain) and their metabolic pathways. These are important research questions as it must be remembered that while of natural origin, PDPs are still viewed by arthropods as xenobiotics, thus having similar targets and metabolic pathways to synthetics. They are consequently not immune from the development of resistance given enough time and over use.

Deciphering the modes of action of PDPs allows the grouping of these products based on their metabolic targets in a similar manner to synthetic compounds, facilitating educated decisions regarding which will be most effective based on knowledge of the resistance/susceptibility status of the target pest. McAllister and Adams carried out a study to this effect testing the efficacy of thymoquinone, nootkatone, and carvacrol on colony strains of *Anopheles gambiae* harbouring known mutations at three different target sites: the sodium channel para-locus mutation (*L1014F KDR*), the acetylcholinesterase (*AChE-1*) gene and a γ-aminobutyric acid receptor mutation of the *Rdl* locus, that confer pyrethrin, organophosphate/carbamate and dieldrin resistance, respectively [101]. A significant difference in the lethal doses between strains was not observed indicating their target sites to be different to those assessed and thus supporting that these products could be effectively employed as control measures to strains harbouring these resistance associated mutations. The inability of nootkatone and carvacrol to cause significant inhibition of acetylcholinesterase has also been confirmed by the work of Anderson and Coates in several important arthropod pests [102]. The same laboratory has provided evidence to suggest that the mode of action of carvacrol may, however, be via the non-competitive inhibition of agonist binding to the nicotine-acetylcholine receptor [103]. In contrast, several components of cashew nut shell liquid, namely anacardic acid, cardol and cardinal, have been shown to possess larvicidal activity against the yellow fever mosquito *Aedes aegypti* with a mode of action involving inhibition of acetylcholinesterase [104]. Inhibition of acetylcholinesterease has also been suggested as a possible mode of action for the anti-feeding effect of ethanolic senescent leaf extracts of *Jatropha gossypifolia* and *Melia azedarach* in larvae of *Spodoptera frugiperda*[105].

The studies described above illustrate the importance of identifying the target site proteins of PDPs in ascertaining their effectiveness, but a clear understanding of PDP detoxification pathways is also important. In this regard synergistic studies with compounds such as piperonyl butoxide, triphenyl phosphate and diethylmaleate, which are known to inhibit the activities cytochrome P450s, carboxylesterases, and glutathione S-transferases, respectively, can provide clues to the detoxification mechanism of PDPs. Several studies have shown that co-administration of piperonyl butoxide with various PDPs, including thymol, eugenol and pyrethum increases the toxicity of these compounds to a range of arthropods [106-109]. In a study by Yadav and colleagues, co-administration with pacholli essential oil decreased the LD_50_ from >250 ppm to 50 ppm, making it almost as effective as basil oil (the most toxic oil tested) without the presence of a synergist [106]. These studies provide evidence that cytochrome P450s can play an active role in the detoxification of PDPs, also demonstrating the potential of previously labelled “non-effective” PDPs if co-administered with a synergist. As changes in the activity of cytochrome P450s, and indeed Glutathione S-transferases, have been identified as a mechanism underlying pesticide resistance, PDPs detoxified by this route may be relatively ineffective in targeting pests that have already developed resistance to synthetics through changes in these enzymes [110,111].

A greater understanding of detoxification pathways and the enzymes involved will allow a more targeted approach to be adopted for the use of PDPs, also providing further avenues to be exploited. In a recent study investigating the effect of *Mintostachy verticillate* essential oil on the house fly *Musca domestica*, researchers discovered that co-administration with piperonyl butoxide resulted in decreased toxicity, indicating that metabolism by a cytochrome P450 was essential for the effectiveness of this essential oil [112]. It was found that menthofuran, a metabolite of the parental compounds (4R)(+)-pulegone and menthone was the active, toxic agent. Thus it may be possible to use detoxification pathways in an advantageous way to identify a range of PDPs that can be used on pesticide resistant arthropod strains, similar to the strategy used in chemotherapy where pro-drugs are used to target specific cancers that overexpress drug metabolising enzymes. Additionally, it may be possible to increase the toxicity of essential oils, such as that of *Mintostachy verticillate*, further by inducing the expression of detoxification enzymes. The ability of compounds to induce the expression of Phase I and II detoxification enzymes in mammals is well understood, but unfortunately this is not the case for arthropods. There is evidence that PDPs may modulate the expression levels of these enzymes as revealed by studies investigating the chemo-preventive effects of neem leaves/flowers and other PDPs in various animal models [113,114]. In these studies neem extracts were found to decrease cytochrome P450 expression, but increase glutathione S-transferases and other Phase II detoxification enzymes, whereas feeding rats sweet basil induced both Phase I and II enzymes.

A final future potential for PDPs lies not in their potential as toxic compounds directly, but in their possible use as synergists or co-administered compounds. In this situation the activity of most interest is their inhibition of detoxification enzymes. In a study by Joffe and colleagues, parsley seed oil and dillapiole oil where found to inhibit cytochrome P450 activity, but not esterase activity, providing a synergistic effect for pyrethrum on *Musca domestica*[108]. In mosquitos, several groups have reported on PDPs and inhibition: plant flavanoids are potent inhibitors of CYP6Z2; thymol and eugenol appear to inhibit both cytochrome P450 and Glutatione S-transferase activities; and essential oils of several native Columbian plants that possess repellent activity are inhibitors of cytochrome P450 activity [115-117].

## Conclusions

The potential of PDPs to target pests of veterinary and medical significance appears strong. Several PDPs are already widely used to this end, with the effectiveness of products such as pyrethrum and neem particularly well supported through a history of successful use against a range of pests in all sectors. Supported by this, the market for PDPs appears to be expanding, particularly for established products. The market for neem-based pesticides, for example, is reportedly growing by nearly 10% per year, with supply able to comfortably meet demand for the foreseeable future [118]. Even for established PDPs, however, the current share of the market occupied in comparison to synthetics remains relatively small [81].

In the on-going search for alternatives to synthetic active ingredients, more 'novel’ PDPs, such as essential oils and their varied chemical components, may hold similar promise to more established products and have been shown to target pests in a number of ways. For many of these products more work is still needed to confirm efficacy, safety and modes of action, addressing limitations such as minimal residual activity with advances in fields such as slow-release technology. A selection of these 'novel’ PDPs has already advanced to commercialisation, perhaps prematurely in some cases as supported by their evidenced risk as veterinary products. Molecular based research, centred around the identification of targets and detoxification pathways/enzymes, could help greatly in ensuring product safety and efficacy, allowing those PDPs with novel arthropod-specific modes of action to be promoted over those with more general activity that target similar pathways to existing synthetics. Such research could also greatly advance combined use of PDPs, with either each other or synthetic compounds.

## Abbreviations

PDP: Plant-derived product; IMM: Integrated mosquito management; DEET: *N*,*N*-Diethyl-*meta*-toluamide; AChE: Acetylecholinestrase; DNA: Deoxyribonucleic acid; GABA: Gamma-aminobutyric acid; EPA: Environmental Protection Agency.

## Competing interests

The authors declared that they have no competing interests.

## Authors’ contributions

DRG conceived the topic and prepared initial drafts of Sections 1, 2 and 3. RDF prepared the initial draft of Section 5. KMG ran literature searches for all sections, collated data on previous reviews for presentation and prepared the initial drafts of Section 6. OAES prepared the initial draft of Section 4. All authors read, critically reviewed and approved an initial version of the manuscript prior to submission. All authors read and approved the final version of the manuscript.
